# Methylomic Signature and Epigenetic Damage Modulation of Bronte Pistachio (*Pistacia vera* L.) Hydrophilic Extract in Differentiated CaCo-2 Cells

**DOI:** 10.3390/nu17162678

**Published:** 2025-08-19

**Authors:** Ilenia Cruciata, Flores Naselli, Sara Volpes, Paola Sofia Cardinale, Laura Greco, Federico Martinelli, Matteo Ramazzotti, Anna Perrone, Graziella Serio, Carla Gentile, Fabio Caradonna

**Affiliations:** 1 Department of Biological, Chemical and Pharmaceutical Sciences and Technologies (STeBiCeF), University of Palermo, Viale delle Scienze, Building 16, 90128 Palermo, Italy; ilenia.cruciata@unipa.it (I.C.); sara.volpes@unipa.it (S.V.); paolasofia.cardinale@unipa.it (P.S.C.); anna-perrone@libero.it (A.P.); graziella.serio01@unipa.it (G.S.); carla.gentile@unipa.it (C.G.); fabio.caradonna@unipa.it (F.C.); 2 Department of Earth and Marine Science (DiSTEM), University of Palermo, Viale delle Scienze, Building 16, 90128 Palermo, Italy; laura.greco08@unipa.it; 3 Department of Biology, University of Florence, Via Madonna del Piano 6, Sesto Fiorentino, 50019 Florence, Italy; federico.martinelli@unifi.it; 4 Department of Experimental and Clinical Biomedical Sciences “Mario Serio”, University of Florence, Viale G.B. Morgagni 50, 50134 Florence, Italy; matteo.ramazzotti@unifi.it; 5 Fondazione Umberto Veronesi ETS, 20121 Milan, Italy; 6 NBFC, National Biodiversity Future Center, 90133 Palermo, Italy

**Keywords:** methylomic signature, DNA methylation, epigenetics, nutrigenomics, CaCo-2 cells

## Abstract

**Background/Objectives**: Nutrigenomics explores how dietary components influence genome function, especially via epigenetic mechanisms like DNA methylation. A key challenge is identifying healthy food-derived molecules capable of counteracting epigenetic damage from harmful dietary elements. Pistachio nuts (*Pistacia vera* L.), particularly the Bronte variety from Sicily, are rich in antioxidant polyphenols. In this study we used a methylomic approach to assess the nutrigenomic potential of a hydrophilic extract from Bronte pistachio (BPHE) in a model of human intestinal epithelium, as well as its capacity to modulate arsenic (As)-induced epigenotoxicity. **Methods**: BPHE was obtained via ethanol/water Soxhlet extraction. CaCo-2 cells were treated with BPHE, alone and after exposure to sodium arsenite. The methylation pattern of the genomic DNA was assessed by methylation-sensitive arbitrarily primed PCR and the methylomic signature was defined by Next-generation bisulfite sequencing. **Results**: BPHE alone did not alter DNA methylation pattern but, at the highest dose, modulated the changes induced by As. The identification of differentially methylated gene promoters in cell treatment vs. untreated controls revealed that BPHE and As primarily induced hyper-methylation, with a synergistic effect when combined. In particular, all the treatments increased methylation levels of gene categories such as pseudogenes, key genes of specific pathways, genes for zinc-finger proteins, homeobox proteins, kinases, antisense RNA, and miRNA. Notably, in co-treatment with As, BPHE promoted hypo-methylation of genes involved in tumor suppression, detoxification, mitochondrial function, and cell division. **Conclusions**: These findings suggest that Bronte pistachio polyphenols may epigenetically steer gene expression toward a protective profile, reducing risks of genomic instability and disease. This supports their potential as nutraceuticals to counter harmful epigenetic effects of toxic food components like arsenic.

## 1. Introduction

Nutrigenomics is a rapidly developing branch of research that explores how dietary components influence genome function, with a particular focus on gene expression regulated by epigenetic mechanisms such as DNA methylation and histone modifications [1]. Within the framework of nutrient–DNA interactions, nutrigenomics considers food-derived molecules as key modulators of gene expression, often investigated through genome-wide approaches. In contrast, nutrigenetics focuses on individual genetic variations (e.g., allelic polymorphisms) and their influence on how the body metabolizes and responds to nutrients [2].

Most of the previous nutrigenomic studies have focused on human nutrition and the effects of nutrients on disease etiology [3], mainly in relation to a specific alimentary regimen. The Mediterranean diet (MD) has been the object of these approaches [4]. The human health benefits associated with a reduction in the risk of developing non-communicable diseases, such as type 2 diabetes, cardiovascular diseases, some neurodegenerative diseases, and cancers, are well known [5]. Previous nutrigenomic studies demonstrated that indicaxanthin, a phytochemical highly concentrated in the edible fruits of the Mediterranean cactus *Opuntia ficus-indica* and with known anti-proliferative activity [6], when administrated at dietary doses to human intestinal epithelial cells, is able to specifically reduce DNA hyper-methylation and restore the expression of key silenced cancer-related genes [7], as well as affect the autophagy process [8]. 

Over the past decade, a wealth of experimental data have significantly reshaped our understanding of the role of nuts in healthy dietary patterns, including the MD. This renewed emphasis on nuts stems from their potential positive impact on serum lipid profiles when incorporated into our diet [9]. Among the 11 species within the *Pistacia* genus, pistachio (*Pistacia vera* L.) stands out as the exclusive producer of an edible nut with substantial market value. While less studied than other nuts like almonds and walnuts, an accumulating body of evidence indicates that pistachio nuts offer not only significant nutritional benefits but also hold great promise as a nutraceutical resource. They boast a rich reservoir of polyphenolic components and have earned a place among the top 50 food products recognized for their exceptional antioxidant potential [10]. Indeed, the well-established health-protective effects attributed to pistachio consumption, combined with its favorable lipid profile, can be attributed to the high concentration of bioactive phytochemicals.

Pistachio cultivation thrives across the Mediterranean region and has gained immense popularity in the United States. In fact, California now stands as the world’s leading pistachio producer. Conversely, pistachio production in Italy remains quite limited, with a primary focus in Sicily, renowned for its exceptional Bronte variety, cultivated in the Etna region. The remarkable quality of this pistachio variety is attributed not only to its distinctive sensory characteristics but also to its significantly higher content of antioxidant polyphenols when compared to other pistachio varieties with more substantial commercial presence [11].

Furthermore, some of our previous research has demonstrated the strong antioxidant potential of a hydroalcoholic extract derived from Bronte pistachio, rich in polyphenols and proanthocyanidins [12]. This extract has exhibited notable antioxidant capabilities in various experimental models and has demonstrated anti-inflammatory effects by modulating the NF-κB activation pathway. Specifically, it has effectively inhibited the inflammatory response triggered by LPS (lipopolysaccharide) or inflammatory cytokines in both macrophages and intestinal epithelial cells [13,14].

A new challenge of modern research in nutrigenomics is to find diet-contained molecules that not only have healthy effects at the metabolic level but are also able to modulate the epigenetic damage induced by other non-beneficial food components consumed through diet. In our hypothesis, their simultaneous or subsequent presence in the same metabolic district of the organism can provide the first molecule with the epigenetic possibility of modulating the damage induced by the second, causing an ameliorative final effect. Dietary antioxidants, particularly polyphenols, are among these beneficial molecules with nutrigenomic modulation capabilities; by reducing ROS levels, they help protect DNA from oxidative damage and support the normalization of DNA methylation [2]. This behavior has already been demonstrated by our group using stilbenoids [15]. This usually occurs in the digestive tract when a subject observes a varied diet. Unfortunately, this is an under-studied aspect considering that we are all subjected to continuous insults by the pollutants contained in food, but also by natural poisons. For example, extremely small concentrations of arsenic in drinking water have been linked to many human cancers [16,17,18], given that they play a role in amplifying the genotoxicity of other mutagenic carcinogens, including ultraviolet radiations [19].

This work intends to investigate, with a methylomic approach, the nutrigenomic property of a Bronte pistachio hydrophilic extract (BPHE) and its mechanisms of action at the epigenetic level in differentiated CaCo-2 cells as a model of the human intestinal epithelium. In fact, it is known that the methylation status of a DNA segment or gene is a putative prodromic condition of its function/expression and represents a basal platform for further functional studies. In addition, we aimed at evaluating the in vitro BPHE ability to modulate and reduce the (epi)genotoxic effect of arsenic (As) at DNA methylation level. Given the absence of studies dealing with combined treatments, this study will advance knowledge on such treatment approach.

## 2. Materials and Methods

### 2.1. Cell Model

The CaCo-2 cell line (ATCC code: HTB-37, Palo Alto, CA, USA), derived from human colon adenocarcinoma, has largely been used as a study model for colon cancer processes and development and also for drug and intestinal disease studies due to their ability to mimic various aspects of intestinal physiology, as well as provide insights into the pathology of the intestinal epithelium. Moreover, CaCo-2 cells, cultured as a monolayer, have the ability to spontaneously undergo differentiation into epithelium-like cells. Once reaching confluence and establishing contact inhibition, cell-to-cell contact and communication generate a physical cue for the cells to differentiate, taking 21 days to reach full differentiation [20]. Differentiated CaCo-2 cells constitute a polarized monolayer characterized by domes, with microvilli on the apical side and tight junctions between adjacent cells, and they represent a model of the human intestinal epithelium for studying the paracellular movement of substances across a monolayer [15].

In the present study, the CaCo-2 cell line was cultured as previously described [21], and the Caco-2 cell monolayer was employed as an in vitro intestinal system to investigate its interaction with potential bioactive compounds derived from the diet.

### 2.2. Plant Material

*Pistacia vera* L. nuts of the Bronte variety were supplied by Pistacchio dell’Etna Srl, located in Bronte, Italy. The seeds were stored in a dark environment at a temperature of 4 °C before extraction.

### 2.3. Extraction Protocol

The seeds were shelled, placed in a −80 °C freezer for 48 h, and then the kernels, along with their skin, were powdered using a mortar. Subsequently, 5 g samples were extracted by a 4 h Soxhlet extraction process using a 70:30 (*v*/*v*) ethanol:water solution with a 1:70 (*w*/*v*) extraction ratio.

Following a clean-up step by centrifugation (10 min at 10,000× *g*, 4 °C) and filtration through a Millex HV 0.45 μm filter (Millipore, Billerica, MA, USA), the extract was collected and stored at −80 °C for future use and analyzed within a span of 5 months. The extraction process was carried out in triplicate.

### 2.4. Content of Polyphenolic Compounds and Antioxidant Activity of BPHE

#### 2.4.1. Total Polyphenol Content

The total polyphenol content (TPC) was assessed using the Folin–Ciocalteu method [22], following previously established procedures [23]. Gallic acid (GA) served as the standard for calibration curve. The results were quantified and expressed as milligrams of GA equivalents (GAE) per 100 g of fresh weight (FW). Each measurement was conducted in triplicate.

#### 2.4.2. Total Proanthocyanidin Content

The total proanthocyanidin content (PAC) was assessed using the DMAC colorimetric assay, a modified version of the method described by Porter et al. (1985) [24]. The method involves the conversion of proanthocyanidins to anthocyanidins through acid hydrolysis in the presence of iron ions and heating the reaction mixture. The assay was performed as previously described [12]. To subtract the contribution of natural anthocyanins in the sample, the sample was processed also in ice instead of by warming. Cyanidin chloride (CC) was used as the standard and the results are expressed as milligrams CC equivalents (CCE) per 100 g of FW. All measurements were conducted in triplicate.

#### 2.4.3. Antioxidant Activity

Antioxidant activity (AA) was assessed using the ABTS assay [25]. The radical ABTS+ was generated by mixing a 7 mM ABTS stock solution with 2.45 mM K_2_S_2_O_8_ and allowing the mixture to stand in the dark at room temperature for 16 h before use. The assay procedure followed previously established protocols [23,26]. Trolox was employed as a reference standard, and the antioxidant activity was quantified and expressed as millimoles of Trolox equivalent (TE) per 100 g of FW. The experiments were replicated three times.

### 2.5. Cell Culture, Treatments, and Positive Controls

Differentiated CaCo-2 cells were treated with BPHE for 3 h mimicking the average time of in vivo intestinal absorption. BPHE was applied at three concentrations—10, 20, and 30 µg GAE/mL of cell culture medium, corresponding approximately to 60, 120, and 180 µM GAE, which are commonly used to evaluate the biological activity of polyphenol-rich extracts in cell models [27]. These concentrations also correspond to about 1.4–4.3 mg of fresh pistachio per mL of culture medium, consistent with the range studied in previous research on the anti-inflammatory effects of Bronte pistachio extract in differentiated CaCo-2 cells exposed to IL-1β [14]. Importantly, these concentrations are below the levels likely to be found in the intestinal lumen after consuming a typical 28 g serving of pistachios, assuming an average gastrointestinal fluid volume of roughly 600 mL. A single extraction batch of BPHE was used for all cell culture treatments.

To simulate an (epi)genotoxic insult, CaCo-2 cells were exposed to sodium arsenite (NaAsO_2_, 10 µg/L of cell culture medium) for 72 h. This concentration is equivalent to the limit of arsenic in drinking water recommended by the World Health Organization’s guidelines [28]. The 72 h incubation period, as previously employed by Volpes et al. (2023) [15] in a similar study, was necessary to reveal the significant differences in DNA methylation, allowing for at least two cycles of cell replication. Three hours before the end of the As treatment, the cells were co-incubated with each concentration of BPHE.

As epigenotoxic positive control, the cells were exposed to 5-azacytidine (5-AzaC, 10 μM), a widely known molecule that acts preferentially on the complex DNMTs-S-adenosine methionine (SAM) affecting genomic DNA methylation status, for 48 h (with fresh addition at 24 h due to the low half-life of this molecule) [21]. Three hours before the end of the 5-AzaC treatment, the cells were co-incubated with each concentration of BPHE. BPHE, NaAsO_2_, and 5-AzaC were dissolved in cell culture medium at the desired concentration. Untreated cells were used as the negative control and are referred to as CTRL. The plan for all of the performed treatments is summarized in Table 1.

### 2.6. Genomic DNA Isolation

Isolation of genomic DNA from cells was carried out with the PureLink Genomic DNA Kit (Invitrogen, Renfrewshire, UK) as previously described [15] and DNAzol (Invitrogen^®^) following the manufacturer’s recommendations. The obtained DNA was quantified using a NanoDrop^®^ ND-1000 (Thermo Fisher Scientific, Wilmington, NC, USA).

### 2.7. MeSAP-PCR-Based Assessment of Genomic DNA Methylation

To assess the genome-wide changes in DNA methylation status possibly induced by each (co-)treatment, methylation-sensitive arbitrarily primed PCR (MeSAP-PCR) assays were performed, as previously described [29], on genomic DNA extracted from all cell culture conditions (Table 1). Briefly, this assay consists of a PCR amplification using an arbitrary primer (5’-AACTGAAGCAGTGGCCTCGCG-3’), in which genomic DNA, previously endonuclease-digested, was used as a template. In particular, genomic DNA was firstly digested by *RsaI* restriction endonuclease (single-digested DNA, SD DNA) and then half the amount of the SD DNA was further digested by *HpaII* methylation-sensitive restriction endonuclease (double-digested DNA, DD DNA). The DNA fingerprinting, obtained by polyacrylamide gel (6%) electrophoresis of the PCR products, was subjected to a densitometric scanning and analyzed using SigmaGel software 1.0 (Jandel Scientific, San Rafael, CA, USA). The SD and DD scans were overlapped to compare any differences between them in terms of disappeared/appeared or intensified/attenuated bands. The greater the number of variations in the band pattern between the SD and DD DNA, the greater the degree of demethylation of the genomic DNA.

### 2.8. Omic Analyses

#### 2.8.1. Illumina Sequencing and Bioinformatics Processing

The TruSeq Methyl Capture EPIC Library Prep Kit (Illumina, San Diego, CA, USA) was used for library preparation following the manufacturer’s instructions. Genomic DNA samples were quantified by Qubit 2.0 Fluorometer (Invitrogen, Carlsbad, CA, USA). Final libraries were checked with both a Qubit 2.0 Fluorometer (Invitrogen, Carlsbad, CA, USA) and Agilent Bioanalyzer DNA assay (Santa Clara, CA, USA). Libraries were then prepared for sequencing and sequenced in the paired-end 150 bp mode on a NovaSeq6000 (Illumina, San Diego, CA, USA).

Processing of raw data for both format conversion and de-multiplexing was performed with the Bcl2Fastq 2.20 version of the Illumina pipeline. Raw paired-end sequencing reads were checked for quality using fastQC 0.11.8 (Brabham Institute, Cambridge, UK), and pre-processing was performed with bbduk v. 38.87 (JGI BBtools). Specifically, the pre-processing involved trimming adapters (k = 13, ktrim = r, mink = 11, tpe, tbo) and removing low-quality reads (maq = 10).

#### 2.8.2. Methylation Analysis from Bisulfite-Sequencing

Methylation signals were obtained from duplicate analysis of next-generation sequencing (NGS) reads using Bismark v.0.22.3 [30]. Bowtie2 v. 2.3.5 [31] was used to map reads to the Bismark-prepared human genome (Homo sapiens GRCh38 primary assembly from ensembl.org). Alignments in the BAM format were sorted with samtools v. 1.10–3 [32]. Methylation signals were analyzed using the R packages methylKit [33], genomation [34], and Granges [35] with gene coordinates taken from the GenCode EBI ftp repository (human release 38). Promoters were defined as regions of 1000 bp before the gene transcription start. Transcription start sites (TSSs) were defined as small regions of 100 bp around the transcription start. Differential analyses between the aforementioned genomic regions (promoters and TSSs) in cells subjected to different treatments were performed using the calculateDiffMeth function of the MethylKit v. 0.99.2, retaining regions showing an adjusted *p*-value ≤ 0.01 and a difference in methylation ≥25%. All statistical analyses were also performed using Wilcoxon signed-rank test as previously reported [36]. Subsequently, the validation of the methylation data was checked using the bisulfite-specific pyrosequencing.

#### 2.8.3. Intersection Analysis

Differentially methylated regions in the different treatments were compared through intersection analyses using the Venn command of the R package gplots v. 3.2.0 and with other R-base functions such as “table” and “grep”. Among the various possible identifiers for genes, gene symbols were chosen as default identifiers for the analysis.

#### 2.8.4. Functional Analysis

Functional inference of differentially methylated regions were performed by an over-representation analysis using the R package clusterProfiler v3.13.0 [37] using pre-formed gene sets obtained from the Molecular Signature Database (MsigDb v. 7.4, Broad Institute, Cambridge, MA, USA) [38,39].

### 2.9. Analysis of Variation in Methylation in Some Structural/Functional Categories of Genes

From the general tables of genes showing a significant variation in the methylation status of their promoters, those belonging to some particularly important categories of structural and functional terms for the cells were extracted. The gene categories chosen are the following: pseudogenes, genes for zinc-finger proteins, genes for antisense RNA, genes for homeobox proteins, genes for kinases, and, lastly, genes for key crucial pathways of the cells, designated by us as “pathway-key genes (PKGs)” and synthetically defined as all genes that are crucial in fundamental pathways of the cell, such as basal metabolism, DNA repair, antioxidant activity, and support for fundamental genetic mechanisms. With these categories of specific targets, a complex overlapped histogram with two vertical axes and several colors were built to gain an integrated visual scenario regarding which and how structural/functional categories of genes vary their methylation under treatments and co-treatments. Moreover, all PKGs were compiled in a table with their proper official acronym, name, and specification of function, according to the literature.

## 3. Results

### 3.1. Content of Polyphenols and Radical Scavenging Activity of BPHE

The total polyphenol content (TPC) of BPHE was assessed using the Folin–Ciocalteu assay, while the radical scavenging action was estimated by the ABTS assay, a commonly used method for evaluating the antioxidant activity (AA) of plant-derived samples.

Since condensed tannins are particularly abundant in the hydrophilic extracts of pistachio and are responsible for the anti-inflammatory properties associated with these extracts, the total proanthocyanidin content (PAC) within the polyphenolic fraction was estimated using the DMAC colorimetric assay.

The TPC, PAC, and AA values of the BPHE are reported in Table 2.

### 3.2. PCR-Based Assessment of BPHE-Induced Genomic DNA Methylation

**Figure 1:**
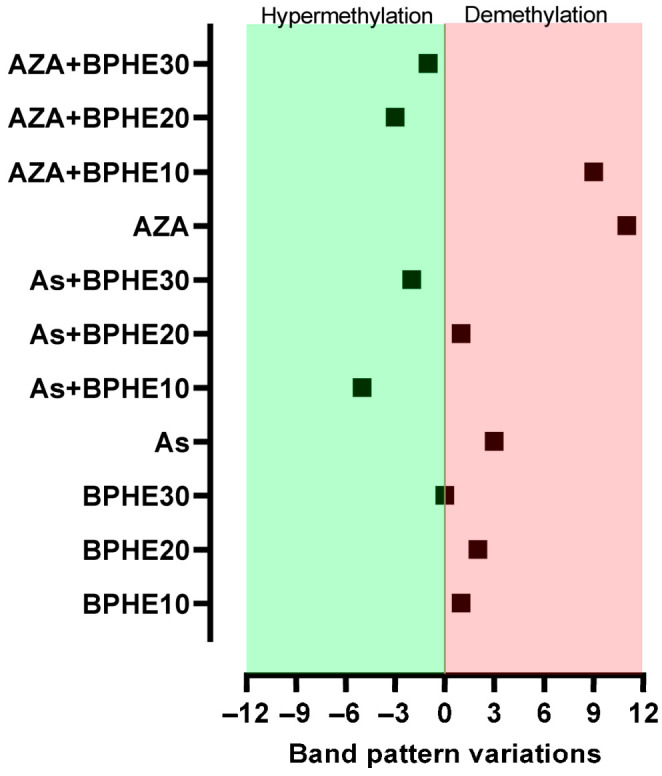
Number of band pattern variations detected by MeSAP-PCR under the cell culture conditions reported in Table 1. The value for each sample represents the difference between the number of band pattern variations in each (co)-treatment and that of untreated cells.

MeSAP-PCR was performed to assess the changes in the genomic DNA methylation status possibly induced by each co-treatment compared to untreated CaCo-2 cells. The PCR carried out in this assay uses an arbitrary primer with a 3’ tail “CGCG”, which directs the amplification toward the CpG-rich sequences, such as those located in gene promoters. The DNA fingerprinting obtained from each condition of cells (co-)treatment was compared with the band pattern obtained from untreated cells (CTRL) to determine the number of variations, in terms of disappeared/appeared or intensified/attenuated bands. A higher number of band pattern variations in treated cells than in untreated ones denotes a genome-wide demethylation; on the contrary, a lower number of bands in treated cells denotes a more methylated status of the genomic DNA compared to the CTRL. The graph in Figure 1 shows the difference in the band pattern variations between untreated cells and each cell culture condition.

From treatments with the three concentrations of BPHE (i.e., 10, 20, or 30 µg GAE/mL of cell culture medium), no significant variation in the band pattern of MeSAP-PCR was observed (Figure 1). Thus, no epigenomic action by BPHE was observed in the differentiated CaCo-2 cell line.

#### Treatment with BPHE Is Able to Decrease As-Induced DNA Demethylation in CaCo-2 Cells

**Figure 2:**
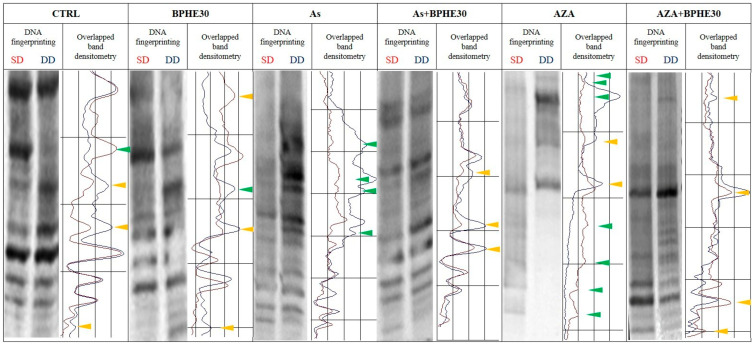
Representative MeSAP-DNA fingerprinting and relative scanning densitometry, indicating genomic DNA methylation of untreated CaCo-2 cells (CTRL) or cells treated with BPHE 30 µg GAE/mL (BPHE30), NaAsO_2_ 10 µg/L (As), NaAsO_2_ 10 µg/L plus BPHE 30 µg GAE/mL (As + BPHE30), 5-AzaC 10 µM (AZA), and 5-AzaC 10 µM plus BPHE 30 µg GAE/mL (AZA + BPHE30). Band pattern variation, in terms of intensification/attenuation (yellow triangles) and appearance/disappearance (green triangles), was evaluated by densitometer scanning of single-digested DNA (SD, profile in red) compared with double-digested DNA (DD, profile in blue).

Treatment with As caused the appearance/disappearance of bands in the DNA fingerprinting of SD and DD DNA relative to this treatment (Figure 2), a band pattern variation more consistent compared to those present in the untreated cells. Thus, the genomic DNA of As-treated cells were substantially demethylated compared to the CTRL condition. By inducing DNA demethylation, As confirmed its epigenotoxic insult in the CaCo-2 cells line [15]. However, when BPHE was added in the combined treatments (As + BPHE10, 20, and 30), it was able to restore a DNA methylation status similar to that of untreated cells or even to induce a hyper-methylation status (Figure 1). Thus, BPHE showed a modulation effect against the epigenetic damage induced by As in CaCo-2 cells.

A similar protective behavior of BPHE, at 20 and 30 µg GAE/mL doses, has been shown toward the significant epigenetic insult by 5-AzaC, used as the demethylating positive control in the combined treatments AZA + BPHE20 and AZA + BPHE30 (Figure 1). Indeed, 5-AzaC-treated cells (AZA) showed nine band pattern variations, most of which involved the appearance/disappearance of bands (Figure 2), showing genome-wide demethylation with respect to the CTRL cells. In the combined treatments, BPHE was able to counteract 5-AzaC-induced DNA demethylation, showing a reduction in band pattern variations with respect to the AZA treatment.

### 3.3. Differential Promoter Methylation Analysis of Treated Cells from Omic Data

In order to exactly locate the position and identity of differential methylation sites (genes) in response to the treatments, we sequenced the DNA of the samples, as well as their counterparts, upon treatment with sodium bisulfite using NGS. Specifically, we investigated the promoter sequences and the TSSs searching for hyper- or hypo-methylation signals, indicating a reprogramming of chromatin accessibility in response to treatments.

Table 3 summarizes the results of the study by reporting the number of genes detected as differentially methylated (hyper-methylated or hypo-methylated) in the promoter regions in the different (co-)treatments with respect to the untreated control cells.

The same results for the number of genes detected as differentially methylated in TSSs are reported in Supplementary File S2.

Our results for the differential methylation of gene promoters between the treated and untreated cells reveal that the most pronounced effect was observed for the treatment with BPHE 30 µg GAE/mL (BPHE30, 745 genes), followed by 5-AzaC (AZA, 705 genes) and sodium arsenite (As, 465 genes) treatments. The co-treatment with BPHE led to a supplement effect under both AZA (823 genes) and As (894 genes) conditions. Overall, we found higher methylation than demethylation effects, stronger in As (377 hyper- vs. 88 hypo-methylated genes) and BPHE30 (614 hyper- vs. 131 hypo-methylated genes) than in AZA (414 hyper- vs. 291 hypo-methylated genes). As expected, 5-AzaC was the strongest demethylating agent; however, when AZA + BPHE30 co-incubation is considered, the number of genes demethylated by 5-AzaC was reduced (from 291 in AZA to 147 in AZA + BPHE30). Treatment with As caused a moderate-to-low variation in the number of genes with different methylation compared to the other treatments, with the majority showing hyper-methylation. However, when As + BPHE30 co-treatment is considered, the number of hyper-methylated genes nearly doubles (from 377 in As to 746 in As + BPHE30), and the number of hypo-methylated genes also increases (from 88 in As to 148 in As + BPHE30) (Table 3).

**Figure 3:**
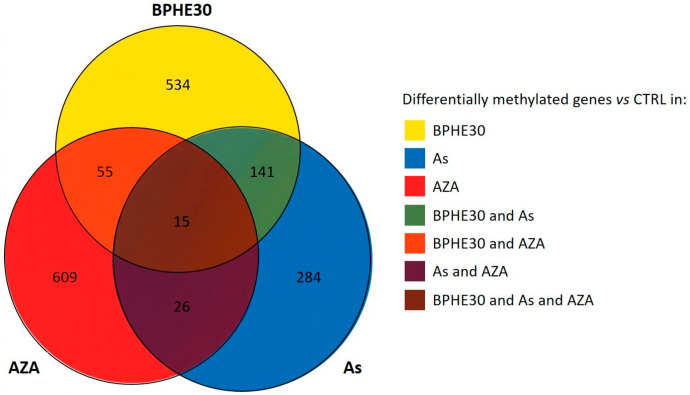
Venn diagram representing the number of genes with evidence of differential methylation under BPHE30, As, or AZA treatment conditions vs. the untreated control.

In Figure 3, the Venn diagram presents the number of genes with differential methylation upon treatment with 5-AzaC, NaAsO_2_, or BPHE 30 µg GAE/mL versus the untreated control. As shown, this intersection analysis allowed for isolation of the specific contributions of the different treatments (see Supplementary File S3 for a complete list of affected genes). We found that the three treatments induced peculiar variations in the methylation levels of the gene promoters, with a slightly higher overlap between BPHE30 and As than between BPHE30 and AZA.

Despite the modest overlap among the three treatments, functional analyses of genes peculiar to the different treatments revealed a common theme related to histone 3 tri-methylation at K27, with potential impacts on cell differentiation and proliferation [40].

**Figure 4:**
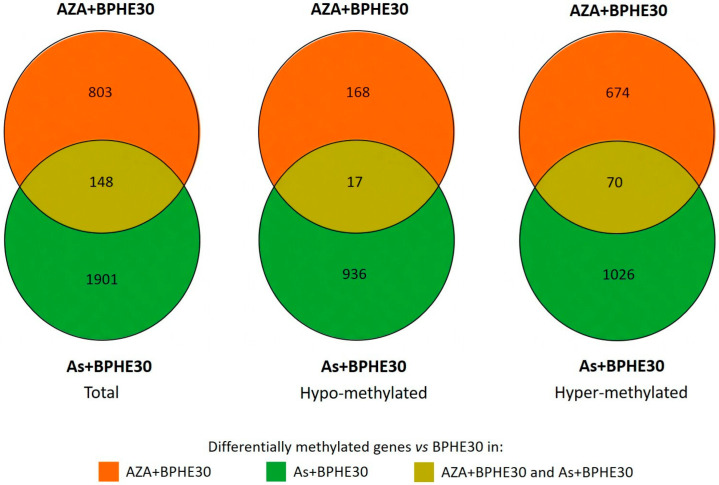
Venn diagrams representing the number of genes with evidence of differential methylation (total, hypo-methylated, or hyper-methylated) in As + BPHE30 and AZA + BPHE30 co-treatments vs. BPHE30 single treatment.

The addition of BPHE 30 μg GAE/mL to NaAsO_2_ or 5-AzaC in the co-treatments (As + BPHE30 and AZA + BPHE, respectively) was further challenged against BPHE alone in order to decouple their effects. The residual pistachio effect was found to be higher (more than double) in As than in AZA and resulted in a large hyper-methylation (four times higher than hypo-methylation) (Figure 4, see Supplementary File S4 for a complete list of affected genes).

### 3.4. Effects of Treatments and Co-Treatments on the Methylation of Structural/Functional Categories of Genes

**Figure 5:**
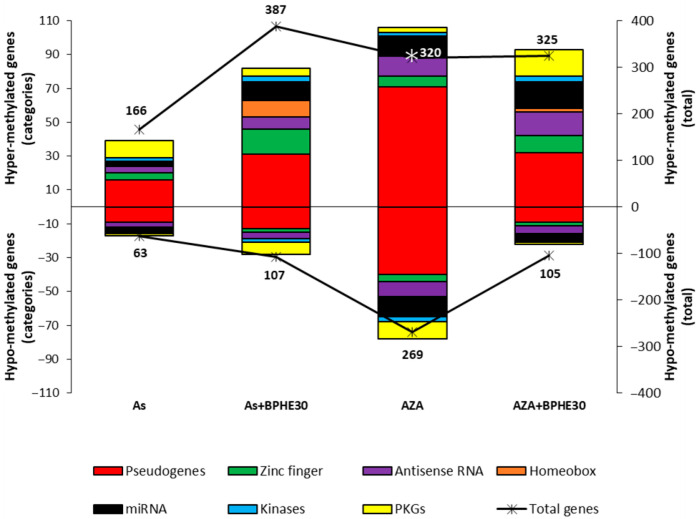
Effects of the treatment with NaAsO_2_ (As) or 5-AzaC (AZA) and of the relative co-treatments with BPHE 30 µg GAE/mL (As + BPHE30 and AZA + BPHE30, respectively) on the methylation status of promoter regions of the structural/functional categories of genes (pseudogenes; genes for zinc-finger proteins, antisense RNA, homeobox proteins, miRNA, and kinases; and pathway-key genes (PKGs)). The chart presents the results as two overlapping graphs. The colored histograms correspond to the left vertical axis, indicating the number of differentially methylated (hyper- or hypo-methylated) genes within each specific structural/functional gene category. The black lines marked with asterisks correspond to the right vertical axis and represent the total number of hyper- or hypo-methylated genes for each individual treatment or co-treatment condition.

We wanted to test, in detail, the variation in the number of differentially methylated promoters, with respect to the untreated control, within some categories of genes in the As and AZA treatments and in their respective co-treatments with BPHE 30 µg GAE/mL (As + BPHE30 and AZA + BPHE30). In particular, pseudogenes; genes for zinc-finger proteins, antisense RNA, homeobox proteins, microRNA, and kinases; and key genes of some pathways (called pathway-key genes, PKGs) were analyzed. The results are shown in Figure 5 and in Table 4, Table 5, Table 6 and Table 7. As shown, all treatments tended to increase methylation levels. Furthermore, most of the methylation variation, regardless of treatment and type of variation induced, is observed in pseudogenes.

This analysis further confirms that 5-AzaC treatment (AZA) causes the most evident increase in demethylation, with 269 hypo-methylated genes recorded including an appreciable quantity of PKGs (Table 4).

When the cells were co-treated with 5-AzaC together with BPHE 30 µg GAE/mL (AZA + BPHE30), the important variations in both the hyper- and hypo-methylating sense already described for the treatment with 5-AzaC alone were reduced. It is noteworthy that the demethylated PKGs from the AZA treatment, as reported in Table 4, were not found among the list of those from the AZA + BPHE30 co-treatment. The PKGs for which the methylation was changed with the AZA + BPHE30 co-treatment are reported in Table 5.

Treatment with sodium arsenite (As) produced an appreciable increase in hyper-methylated PKGs and only one hypo-methylated PKG (Table 6).

Interestingly, when cells are co-treated with NaAsO_2_ together with BPHE 30 µg GAE/mL (As + BPHE30), the variation in differentially methylated genes assumes important numbers recording the maximum of hyper-methylated genes (387). In particular, there was an increase in the number of hyper-methylated genes for zinc-finger proteins, antisense RNA, homeobox proteins, and microRNA. Furthermore, this co-treatment varied the methylation of some PKGs (Table 7).

**Figure 6:**
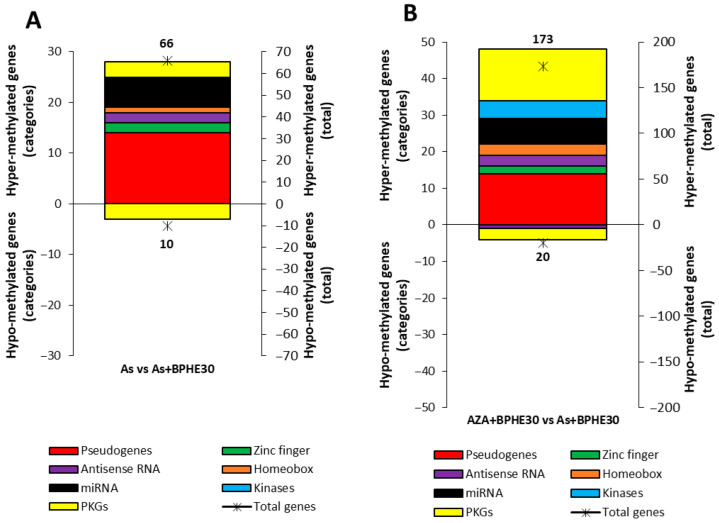
Integrated methylation analysis of promotor regions of the structural/functional categories of genes (pseudogenes; genes for zinc-finger proteins, antisense RNA, homeobox proteins, miRNA, and kinases; and pathway-key genes (PKGs)) between the (**A**) As treatment and As + BPHE30 co-treatment and the (**B**) AZA + BPHE30 and As + BPHE30 co-treatments. The charts present the results as two overlapping graphs. The colored histograms correspond to the left vertical axis, indicating the number of differentially methylated (hyper- or hypo-methylated) genes within each specific structural/functional gene category. The black asterisks correspond to the right vertical axis and represent the total number of hyper- or hypo-methylated genes for each condition.

An integrated comparison was also made between two treatments or co-treatments in order to highlight some methylation variations in noteworthy genes. In particular, Figure 6 shows the number of variations in differentially methylated genes within structural/functional categories by comparing between the treatments As and As + BPHE30 and also between the co-treatments AZA + BPHE30 and As + BHPE30.

The PKGs detected by an integrated comparison between the As and As + BPHE30 treatments are reported in Table 8.

The PKGs detected by an integrated comparison between the AZA + BPHE30 and As + BPHE30 co-treatments are reported in Table 9.

## 4. Discussion

Nutrigenomics, a modern branch of genetics and genomics, investigates how dietary components influence gene expression through epigenetic mechanisms. Understanding whether bioactive food molecules can exert beneficial effects—complementing conventional therapies—may offer added value in enhancing therapeutic outcomes with minimal additional effort. This becomes particularly relevant when such molecules exhibit genotoxicity-modulating properties, potentially counteracting the damage caused by harmful dietary components. In such cases, the same molecule may provide dual benefits by both supporting genome stability and mitigating genotoxic insults. Among various dietary elements, we focused on the Bronte pistachio (*Pistacia vera* L. var. Bronte), which represents a peculiarity of the Mediterranean diet, a plant that grows in the foothills region of the Etna volcano (Southern Italy). In a previous study, we carried out a comprehensive qualitative and quantitative chemical characterization of the phenolic compounds and fatty acids of this variety of *Pistacia vera* L. [11]. To assess the bioactive molecules content and the antioxidant properties of a hydrophilic extract obtained from Bronte pistachio (BPHE), we preliminarily evaluated its total polyphenol content (TPC) and its radical scavenging activity using an ABTS assay. Considering the known bioactivity of the proanthocyanidin fraction of hydrophilic pistachio extracts in experimental models [13,14], we also estimated the total proanthocyanidin content (PAC). The results reveal extremely high levels of antioxidant polyphenols in our BPHE (Table 2). Soxhlet extraction with an ethanol/water mixture (70:30) extracted polyphenols in quantities four times greater than those obtained using the maceration method with a methanol/water mixture (70:30), as reported in the literature [12], and this method also resulted in an extract with superior antioxidant activity. BPHE demonstrates superior radical scavenging activity, as indicated by a higher ABTS value, compared to the extract obtained through maceration with methanol/water. This suggests that Soxhlet extraction not only improves the yield of polyphenols but also maintains, or potentially enhances, their antioxidant efficacy. We based our evaluation on total phenolic content (TPC) and proanthocyanidins (PACs), which are widely recognized in the literature as major contributors to epigenetic modulation [109,110]. These compounds influence chromatin accessibility and the activity of epigenetic enzymes, such as DNMTs and HDACs. Thus, we evaluated the nutrigenomic effect and the ability to modulate genotoxic damage by BPHE, and we did so according to two parallel methods applied to human enterocytes (differentiated CaCo-2 cells). Notably the concentrations of BPHE used here support the physiological relevance of our findings.

At first, we used an epigenetic approach in order to evaluate the DNA methylation status of cells treated with three different concentrations of BPHE compared to molecules with a known epigenotoxic effect on DNA methylation; subsequently, we determined the methylome of the same cells, choosing the higher dose of BPHE, with an *omic* approach. In particular, we performed co-treatments with BPHE and arsenic, normally contained in drinking water, to investigate whether the pistachio extract also has the ability to modulate genotoxicity.

In order to evaluate the effect of BPHE, alone or in combination with genotoxic molecules (As or 5-AzaC), on DNA methylation, we preliminary used MeSAP-PCR. This assay is able to define DNA methylation status at a genome-wide level, although it is directed, in particular, toward the CpG islands thanks to the 3’ tail “CGCG” of the arbitrary primer used in the amplification step. As expected, this assay revealed that BPHE (10, 20, and 30 µg GAE/mL of cell culture medium) did not have an effect on the DNA methylation of differentiated CaCo-2 cells, maintaining a condition similar to that of untreated cells; BPHE 30 µg GAE/mL, in particular, resulted in exactly the same methylation condition of the untreated cells (Figure 1). It is known that As induces genomic instability [111], and genomic instability has also been reported to be associated with genomic demethylation of the DNA of unstable cells [112]. Here, the treatment with NaAsO_2_ resulted in a certain degree of demethylation of genomic DNA compared to untreated cells (Figure 1), also configuring As as an epigenotoxic agent, as already demonstrated by us [15]. In the combined treatments, when BPHE was added at the end of the incubation period with As, the DNA demethylation As induced was recovered. Furthermore, BPHE induced hyper-methylation at 10 and 30 µg GAE/mL doses (Figure 1). Thus, BPHE demonstrated the ability to modulate the epigenotoxic damage caused by As in CaCo-2 cells. The known demethylating agent 5-AzaC, used as the epigenotoxic positive control, induced in differentiated CaCo-2 cells a significant DNA demethylation, also in combination with BPHE 10 µg GAE/mL (Figure 1). However, higher BPHE doses added in combination with 5-AzaC induced DNA hyper-methylation. In particular, BPHE 30 µg GAE/mL induced a similar effect on DNA hyper-methylation when added after both As and 5-AzaC (Figure 1). These results suggest a protective effect of Bronte pistachio extract on the insults of dangerous molecules commonly present in our diet, such as arsenic.

To better investigate the epigenetic effect of BPHE and its activity toward DNA methylation alterations, we used an *omic* approach to define the methylomic signature of the differentiated CaCo-2 cell line treated with BPHE, alone or in combination with As or 5-AzaC. For this purpose, we chose only the highest BPHE dose—30 µg GAE/mL—for the cell culture medium, as it was the one for which the MeSAP-PCR showed a DNA methylation status more similar to that of the untreated cells.

As can be seen in Table 3, BPHE changed the methylation status of 745 gene promoters compared to the untreated control cells, most of which were hyper-methylated. Similarly, As mainly showed a hyper-methylating effect on promoter regions of differentiated CaCo-2 cells, although for a smaller number of genes (about 50%) compared to those hyper-methylated by BPHE alone. Previous studies from other laboratories, performed on tumoral CaCo-2 cells, have reported that As has a similar hyper-methylating effect [113]. Since we cannot make a comparison with other data on differentiated CaCo-2 cells, we can only hypothesize that the effect of As on DNA methylation is independent from the condition of genomic instability of CaCo-2 cells. Our data are the first to report on differentiated CaCo-2 cells, considered as normal enterocytes in vitro. Although MeSAP-PCR on As-treated cells has shown a certain degree of DNA demethylation, at the *omic* level, the As treatment resulted in the genome of differentiated CaCo-2 cells having a higher overall number of hyper-methylated regions than that of the control cells. Indeed, as shown in Figure 3 and Figure 4, the As + BPHE30 co-treatment exhibited a synergistic action: the number of hyper-methylated gene promoters increased compared to that of the individual treatments, indicating that, under these specific conditions and in this experimental system, As and BPHE have similar and additive effects. The discrepancy between the results of the MeSAP-PCR and the methylome evaluation, both for the BPHE30 and As treatments, can be attributed to the inherent differences between the two methodologies in terms of resolution, genomic coverage, and sensitivity to methylation patterns. MeSAP-PCR relies on methylation-sensitive restriction enzymes that recognize specific sequences whose cleavage is inhibited by methylation. Consequently, it provides a snapshot of methylation changes at selected genomic loci, often enriched in repetitive or highly methylated regions (e.g., CpG islands), but it lacks nucleotide-level resolution and may miss methylation changes occurring in non-CpG contexts. In contrast, next-generation bisulfite sequencing enables single-base resolution mapping of methylated cytosines across a broader portion of the genome, offering a much more comprehensive view of DNA methylation patterns. Therefore, the methylome fraction assessed by MeSAP-PCR may not accurately represent the entire methylome, as revealed by *omic* analysis, but rather a focus of the methylation status of the genomic CpG islands. Considering this, the observed discrepancies between the two methodologies do not necessarily reflect contradictory biological results but rather highlight the methodological and interpretative differences between targeted enzymatic fingerprinting and genome-wide methylation profiling. Integrating both approaches, as performed in the present study, can provide complementary insights into DNA methylation dynamics, particularly when interpreting complex or subtle epigenetic modifications.

Regarding the AZA treatment, if, on the one hand, 5-AzaC is confirmed as a molecule capable of greatly influencing genomic DNA methylation, on the other hand, it shows its specific and atypical behavior. As it is possible to observe, in this normaloid cellular system, 5-AzaC shows a pleiotropic behavior causing both an increase and decrease in the methylation of gene promoters. However, this action is not perfectly balanced, and it can be said that 5-AzaC induces an increase in methylation in a slightly higher number of genes. Probably, also for 5-AzaC, it is also important to consider the normal-like nature of differentiated CaCo-2 cells, particularly with regard to their baseline level of DNA methylation. Interestingly, when the cells were co-treated with 5-AzaC and BPHE, the number of differentially methylated genes increased with a clear prevalence of hyper-methylated ones. It seems that BPHE enforces the slight hyper-methylating action carried out by 5-AzaC alone.

Even more interesting are the insights that can be drawn from methylome analysis focused on specific structural/functional categories of genes. As shown in Figure 5, the upper part of the figure appears more expanded compared to the lower part, indicating that all the treatments cause a greater increase in the number of hyper-methylated genes than demethylated ones. Moreover, by following the red color, it becomes immediately apparent that most of the methylation changes—regardless of the treatment or the type of variation—are concentrated in pseudogenes. There are currently few contributions in the literature regarding the relationship between methylation of pseudogenes and phenotypic outcomes. While it can be hypothesized that pseudogene methylation plays a role in cell genome managing, assigning biological relevance to this finding is challenging, given that many pseudogenes are known to be not expressed. However, as reported by Kovalenko et al. (2021) [114], some pseudogenes, like *PTENP1*, were transcribed with the aim of forming long noncoding RNAs able to regulate gene expression. In our experiments, we observed a massive modification of the methylation pattern of pseudogenes. This evidence offers fascinating insights into the putative indirect power of dietary molecules that affect not only the known activity of regulating the DNA methylation of specific genes but also the still unknown regulation of gene expression related to pseudogenes. On the other hand, it is important to remember that pseudogenes are non-functional genes and that non-functionality can, in general, be linked to both silencing gene mutations and epigenetic modifications that inhibit expression.

The addition of BPHE in the co-incubation with As increases the number of methylated genes for zinc-finger proteins, homeobox proteins, and miRNA, as well as distributes the number of methylated/demethylated PKGs differently. It is important to remember that DNA methylation is closely associated with miRNA expression, and, in turn, miRNA genes regulate DNA methylation by targeting *DNMT* mRNAs [55]. Moreover, it may be considered that re-expression, out of morphogenesis, of *HOX* genes is associated with different solid tumors and that genes for homeobox proteins are known as widespread transcriptional regulators. In particular, CpG island hyper-methylation in several *HOX* genes contributes to the silencing of DNA repair genes with detrimental effects on genomic stability that can lead to oncogenic transformation, including in colorectal cancer [115]. In this complex scenario, our data demonstrate that BPHE greatly modulates all this gene subset and provides insight into the integration rate of its effects on the entire epigenome.

Regarding the co-treatment with 5-AzaC or As together with BPHE, the most evident data show that the addition of BPHE significantly increases the number of methylated PKGs. Attempting to draw conclusions from this intricate network of methylation and demethylation can be arduous, especially regarding the nutrigenomic role of BPHE. Nevertheless, looking at Table 4, Table 5, Table 6 and Table 7, which report the PKGs for each treatment or co-treatment, some conclusions emerge. It can be noted that 5-AzaC alone demethylated genes of the (i) RAS-family, (ii) histone deacetylase, (iii) Src family kinases (indirectly), and (iv) a protein involved in cell migration, adhesion, and in cytoskeleton management (Table 4). These genes, the expression of which is probably unblocked by 5-AzaC-induced demethylation, can increase genomic instability and could promote a tumoral transformation. Co-treatment AZA + BPHE30, which hyper-methylates three genes implicated in DNA repair, the pentose phosphate pathway, and xenobiotic detoxification, was, notably, also able to putatively block the expression of genes involved in autophagy, genotoxic-stress inducement, genomic instability, tumor pathogenesis, and dysfunction of peroxisomes, as well as promoting hypo-methylation of a gene coding for a cell-cycle regulator (Table 7). In parallel, while As alone hyper-methylated and verisimilarly blocked genes implicated in MEOS detoxification, redox management, autophagy, and assembly of repair DNA polymerase (Table 5), the co-presence of As and BPHE, interestingly, hypo-methylated genes coding a tumor-suppressor gene, cytochrome c oxidase, aldehyde dehydrogenase with known tumor-suppressive properties, and kinesin required for spindle assembly and chromosome movement (Table 6). From both the co-treatments, it can be deduced that the presence of BPHE could epigenetically modulate the gene network expression of differentiated CaCo-2 cells toward a better landscape, decreasing all conditions that are prodromal to genomic instability, cancer, and, in general, to a genotoxic condition. Clearly, further functional gene expression studies have to be performed to move from a hypothesis to the certainty that these methylomic changes are reflected in changes in gene expression.

The *omic* nature of our data also offers the mathematic possibility of conducting an integrated comparison between the two classes of data coming from the treatments or co-treatments to highlight some methylation variations in noteworthy genes. It is possible to overlap the cohorts of data regarding the As treatment and AZA + BPHE30 co-treatment versus the As + BHPE30 co-treatment. Figure 6 shows the number variations in differentially methylated genes within specific structural/functional categories via a comparison between the As treatment and As + BPHE30 and also between the AZA + BPHE30 and As + BHPE30 co-treatments. In Figure 6A, in particular, the comparison of As vs. As + BPHE30 highlights the behavior of BPHE: almost the entire distribution is positioned in the upper part of the histogram, showing that BHPE mainly had a methylating effect. Table 8, in addition, reports that the PKGs involved in changing the methylation in this integrated analysis were all genes not particularly important for enterocyte cell functions. Similarly, Figure 6B, for the comparison of AZA + BPHE30 versus As + BHPE30, highlights the different behaviors of 5-AzaC and As. In addition to the large number of hyper-methylated pseudogenes, it is worth noting that there were a large number, similar to pseudogenes, of hyper-methylated PKGs, greater in number than the corresponding hypo-methylated ones. These results confirm the methylating power of As and underline the ineffectiveness of 5-AzaC in counteracting this effect. Table 8 and Table 9 provide further details regarding the gene activities involved in these integrated analyses; it should be noted that a DNA repair gene and a gene for nucleotide synthesis are hyper-methylated, while genes for cell adhesion and histone deacetylase are hypo-methylated.

Intestinal epithelial cells are naturally exposed to continuous and repetitive stress, often of genotoxic origin, and it is possible that compounds derived from pistachio could exert a compensatory effect that supports cellular health. The present findings suggest that BPHE exerts a protective epigenetic effect against genotoxic insult on differentiated CaCo-2 cells. Although these preliminary in vitro findings are promising, further investigations are needed to confirm this protective effect in vivo and, ideally, in human models. Similar effects could also be explored for other dietary bioactives, particularly those characteristic of the Mediterranean diet. Such research may contribute to a better understanding of the diet’s established protective role against gastrointestinal diseases, including cancer.

## 5. Conclusions

In this study Bronte pistachio nuts were used to obtain a hydrophilic extract (BPHE) particularly rich in polyphenols with a strong antioxidant and radical scavenging activity. Our BPHE was tested in vitro on a model of human intestinal epithelium (differentiated CaCo-2 cells) in order to assess its ability to modulate DNA at the epigenetic level and identify the methylomic signature of genomic DNA after an epigenotoxic insult induced by arsenic.

Both BPHE and As induce a predominantly hyper-methylating effect on gene promoter regions, and, interestingly, their combination exerts a synergistic increase in DNA hyper-methylation, highlighting that BPHE is able to modulate the methylation changes induced by As. A gene category-specific analysis revealed that most of the methylation changes occurred in pseudogenes and that the co-treatment with BPHE and As increased the number of methylated genes for zinc-finger proteins, homeobox proteins, miRNA, and antisense RNA. Regarding PKGs, while As alone hyper-methylates genes implicated in MEOS detoxification, redox managing, autophagy, and assembly of repair DNA polymerase, the co-presence of As and BPHE hypo-methylated a subset of critical genes involved in tumor suppression, detoxification, mitochondrial function, and cell division. These findings suggest that BPHE exerts a protective epigenetic influence on the gene network expression of differentiated CaCo-2 cells, restoring a more balanced methylation landscape and potentially reducing the risk of genomic instability and related pathologies, including cancer.

## Figures and Tables

**Table 1:** Cell treatments plan. BPHE: Bronte pistachio hydrophilic Extract; 5-AzaC: 5-azacytidine; GAE: gallic acid equivalents. The incubation times of the co-treatment with BPHE at various concentrations is indicated in parentheses; BPHE was added during the final hours of the overall treatment period, which lasted 48 or 72 h.

Treatment Acronym	Treatment	Co-Treatment	Incubation Time (h)
BPHE10	BPHE [10 µg GAE/mL]	-	3
BPHE20	BPHE [20 µg GAE/mL]	-	3
BPHE30	BPHE [30 µg GAE/mL]	-	3
As	NaAsO_2_ [10 µg/L]	-	72
As + BPHE10	NaAsO_2_ [10 µg/L]	BPHE [10 µg GAE/mL]	72 (3)
As + BPHE20	NaAsO_2_ [10 µg/L]	BPHE [20 µg GAE/mL]	72 (3)
As + BPHE30	NaAsO_2_ [10 µg/L]	BPHE [30 µg GAE/mL]	72 (3)
AZA	5-AzaC [10 µM]	-	48
AZA + BPHE10	5-AzaC [10 µM]	BPHE [10 µg GAE/mL]	48 (3)
AZA + BPHE20	5-AzaC [10 µM]	BPHE [20 µg GAE/mL]	48 (3)
AZA + BPHE30	5-AzaC [10 µM]	BPHE [30 µg GAE/mL]	48 (3)

**Table 2:** Total polyphenol content (TPC), total proanthocyanidin content (PAC), and antioxidant activity (AA) of BPHE. Values are expressed as the mean ± SD of three experiments carried out in triplicate. GAE: gallic acid equivalents; CCE: cyanidin chloride equivalents; TE: Trolox equivalents; FW: fresh weight.

TPC	704.41 ± 68.14 mg GAE/100 g FW
PAC	132.83 ± 7.23 mg CCE/100 g FW
AA	3.01 ± 0.07 mmol TE/100 g FW

**Table 3:** Number of genes resulting as differentially methylated (hyper- or hypo-methylated) in the promoter regions in the different cell (co-)treatments with respect to the untreated control cells (see Supplementary File S1 for the complete report).

Treatment vs. Untreated Control	Total	Hyper-Methylated	Hypo-Methylated
BPHE30	745	614	131
As	465	377	88
As + BPHE30	894	746	148
AZA	705	414	291
AZA + BPHE30	823	676	147

**Table 4:** Pathway-key genes (PKGs) differentially methylated by 5-AzaC treatment (AZA) in CaCo-2 cells.

	Gene Name	Gene Description	Principal Function of the Gene Product
**Hyper-methylated**	*HSPB7*	Heat-shock protein family B small member 7	Regulation of cytoskeleton integrity [41]
	*CYP26B1*	Cytochrome P450 family 26 subfamily B member 1	Oxidative metabolism of retinoic acid [42]
	*GCK*	Glucokinase	Glucose metabolism [43]
**Hypo-methylated**	*VWA1*	von Willebrand factor A domain-containing 1	Unknown-function orphan extracellular matrix protein [44]
	*RAB25*	RAB25, member RAS oncogene family	Member of Rab-GTPase family implicated in cancer progression [45]
	*SGMS2*	Sphingomyelin synthase 2	Production of sphingomyelin [46]
	*HSPA4L*	Heat-shock protein family A (Hsp70) member 4-like	Fertility-related proteins in testes [47]
	*BHMT2*	Betaine-homocysteine S-methyltransferase 2	Regulation of lipid metabolism in metabolic-associated fatty liver disease pathogenesis [48]
	*CASP8AP2*	Caspase-8-associated protein 2	Regulation of epithelial-to-mesenchymal transition plasticity [49]
	*TH*	Tyrosine hydroxylase	Biosynthesis of dopamine and other catecholamines [50]
	*PTPRCAP*	Protein tyrosine phosphatase receptor-type-C associated protein	Positive regulation of protein tyrosine phosphatase CD45, which activates Src family kinases implicated in tumorigenesis [51]
	*PSTPIP1*	Proline–serine–threonine phosphatase-interacting protein 1	T-cell activation, differentiation, migration, cell adhesion, and cytoskeleton managing [52]
	*MBD3L3*	Methyl-CpG-binding domain protein 3 like-3	Component of the histone deacetylase complex, in regulation of cell-cycle progression and cell death [53]

**Table 5:** Pathway-key genes (PKGs) differentially methylated in AZA + BPHE30 co-treatment in CaCo-2 cells.

	Gene Name	Gene Description	Principal Function of the Gene Product
**Hyper-methylated**	*PGM2*	Phosphoglucomutase 2	Glycolysis and glycogen metabolism [54]
	*SGMS2*	Sphingomyelin synthase 2	Natural killer cell recruitment, immune landscape and genomic instability [55]
	*HCP5*	HLA complex P5	Oncogene-accelerating cancer-cell growth, invasion, metastasis, vascularization, and drug resistance in renal cell carcinoma [56]
	*FNDC1*	Fibronectin type III domain-containing 1	Bone-metabolism-related factor [57]
	*ASL*	Argininosuccinate lyase	Cleavage of argininosuccinic acid to produce arginine and fumarate in the fourth step of the urea cycle [58]
	*NANS*	N-acetylneuraminate synthase	Sialic acid biosynthesis and transportation [59]
	*NDOR1*	NADPH-dependent diflavin oxidoreductase 1	Biogenesis of iron–sulfur cluster proteins [60]
	*HKDC1*	Hexokinase domain-containing 1	Mitochondrial and lysosomal homeostasis [61]
	*TALDO1*	Transaldolase 1	Pentose phosphate pathway [62]
	*SAA1*	Serum amyloid A1	Lipid metabolism, regulation of inflammation, and tumor pathogenesis [63]
	*PLAAT2*	Phospholipase A and acyltransferase 2	Dysfunction of peroxisomes [64]
	*DRAM1*	DNA-damage-regulated autophagy modulator 1	Genotoxic-stress-induced alternative autophagy by closure of isolation membranes downstream of p53 [65]
	*UNG*	Uracil DNA glycosylase	Excision of hypoxanthine from DNA, thus triggering a base excision repair (BER) process [66]
	*DHRS4*	Dehydrogenase/reductase 4	Reduction in compounds containing aldehyde, ketone, and quinone groups, as well in humans, in xenobiotics [67]
	*IGHD3–10*	Immunoglobulin-heavy diversity 3–10	VDJ gene most frequently used in immunoglobulin synthesis [68]
	*TNFSF14*	TNF superfamily member 14	Tumor necrosis superfamily ligand with a broad range of adaptive and innate immune activities [69]
**Hypo-methylated**	*FZD3*	Frizzled class receptor 3	Regulation of cell growth, death, differentiation, and cell cycle [70]

**Table 6:** Pathway-key genes (PKGs) differentially methylated by NaAsO_2_ treatment (As) in CaCo-2 cells.

	Gene Name	Gene Description	Principal Function of the Gene Product
**Hyper-methylated**	*CYP2E1*	Cytochrome P450 family 2 subfamily E member 1	Hepatic MEOS metabolizer enzyme, particularly polymorphic in humans [71]
	*CDK2AP2*	Cyclin-dependent kinase 2 associated protein 2	Regulation of cell cycle and mitosis [72]
	*HSPB2*	Heat-shock protein family B (small) member 2	Redox metabolism with implications in cardiovascular diseases [73]
	*GAPDH*	Glyceraldehyde-3-phosphate dehydrogenase	Aerobic glycolysis [74]
	*CNTN1*	Contactin 1	Cell-adhesion molecules implicated in myelinated axon organization [75]
	*WNT1*	Wnt family member 1	Oncogenesis and cell–cell signals in development [76]
	*HSPB8*	Heat-shock protein family B (small) member 8	Chaperone-assisted selective autophagy [77]
	*MLNR*	Motilin receptor	Regulation of gastrointestinal motility; function not well understood in humans [78]
	*HSPB9*	Heat-shock protein family B (small) member 9	Specifically expressed in testis, notably in the spermatogenic cells, with a sex-related role [79]
	*POLR3F*	RNA polymerase III subunit F	RNA polymerase III polypeptide F [80]
**Hypo-methylated**	*MYO18B*	Myosin XVIIIB	Sarcomere assembly in fast skeletal muscle [81]

**Table 7:** Pathway-key genes (PKGs) differentially methylated in As + BPHE30 co-treatment in CaCo-2 cells.

	Gene Name	Gene Description	Principal Function of the Gene Product
**Hyper-methylated**	*ATP1B1*	ATPase Na^+^/K^+^ transporting subunit beta 1	Regulation of the uptake of solutes and establishment of an appropriate Na^+^ gradient [82]
	*IL15*	Interleukin 15	Inflammation and homeostasis of the immune system [83]
	*CYP26C1*	Cytochrome P450 family 26 subfamily C member 1	Regulation of the concentration of retinoic acid in cells [84]
	*CCNA1*	Cyclin A1	Regulation of the cell cycle [85]
**Hypo-methylated**	*SEMA6C*	Semaphorin 6C	Signaling molecules controlling axonal wiring and embryo development; support of viability and growth of cancer cells [86]
	*ENO4*	Enolase 4	Motility and organization of the sperm flagellum [87]
	*KCNQ1*	Potassium voltage-gated channel subfamily Q member 1	The lncRNA within its gene sequence participates in the pathogenesis of a diversity of cancers as well as non-cancerous conditions [88]
	*MT1M*	Metallothionein 1M	Cysteine-rich cytosolic protein reported to be a tumor suppressor gene in multiple cancers [89]
	*COX4I1*	Cytochrome c oxidase subunit 4I1	Mitochondrial biogenesis [90]
	*ALDH3A2*	Aldehyde dehydrogenase 3 family member A2	Tumor-suppressive role, influencing epithelial–mesenchymal transition [91]
	*KIF2B*	Kinesin family member 2B	Spindle assembly, chromosome movement, and microtubule depolymerase activities [92]

**Table 8:** Pathway-key genes (PKGs) differentially methylated in the integrated comparison between the As treatment and As + BPHE30 co-treatment in CaCo-2 cells.

	Gene Name	Gene Description	Principal Function of the Gene Product
**Hyper-methylated**	*IGHJ5*	Immunoglobulin heavy joining 5	Clonotypes in *IGHV* gene combinations associated with the production of autoantibodies [93]
	*IGHJ4*	Immunoglobulin heavy joining 4	Clonotypes in *IGHV* gene combinations associated with the production of autoantibodies [93]
	*IGHD6–13*	Immunoglobulin heavy diversity 6–13	Clonotypes in *IGHV* gene combinations associated with the production of autoantibodies [93]
**Hypo-methylated**	*POLH*	DNA polymerase eta	DNA repair polymerase involved in post-replication short-patch repair [94]
	*FANK1*	Fibronectin type III and ankyrin repeat domains 1	Nuclear protein exclusively expressed during the transition from the meiotic to the haploid phase of spermatogenesis in testis [95]
	*TRAV22*	Alpha variable 22	T-cell receptor repertoires segment clonotypes [96]

**Table 9:** Pathway-key genes (PKGs) differentially methylated in the integrated comparison between AZA + BPHE30 and As + BPHE30 co-treatments in CaCo-2 cells.

	Gene Name	Gene Description	Principal Function of the Gene Product
**Hyper-methylated**	*TNFRSF25*	TNF receptor superfamily member 25	Immune checkpoint gene [97]
	*LY6G6F*	Lymphocyte antigen 6 family member G6F	Cell-mediated adaptive immunity-related protein [98]
	*PVT1*	Pvt1 oncogene	Protein closely linked to cancer development via microRNAs [96]
	*CTSW*	Cathepsin W	Member of the papain family cysteine proteases, involved in antigen-presenting cells, antigen processing, and immune response control [96]
	*CABP4*	Calcium-binding protein 4	Neuronal-Ca^2+^-binding protein that participates in many cellular processes by regulating the concentration of free Ca^2+^ ions [99]
	*IGHD3–16*	Immunoglobulin heavy diversity 3–16	Clonotypes in *IGHV* gene combinations associated with the production of autoantibodies [93]
	*IGHV3–53*	Immunoglobulin heavy variable 3–53	Clonotypes in *IGHV* gene combinations associated with production of autoantibodies [93]
	*MPG*	N-methylpurine DNA glycosylase	Initiation of base excision repair in DNA by removing alkylated, deaminated, and lipid-peroxidation-induced purine adducts [100]
	*NDUFB10*	NADH ubiquinone oxidoreductase subunit B10	Interacting partner and in vivo target of CHCHD4, a disulfide-bond-forming enzyme [101]
	*DUS2*	Dihydrouridine synthase 2	Flavoenzyme that catalyzes synthesis of dihydrouridine within the complex elbow structure of tRNA [102]
	*CSF3*	Colony-stimulating factor 3	Related to innate and adaptive immune systems [103]
	*CDH4*	Cadherin 4	Differentiation of retina, kidney, striated muscle, and brain nerves [104]
	*COL20A1*	Collagen alpha 1 chain	Collagen synthesis [105]
	*APOL5*	Apolipoprotein L5	Member of APOL lipoprotein family, associated with inflammation, autophagy, and kidney disease [106]
**Hypo-methylated**	*HAT1*	Histone acetyltransferase 1	Acetylation of newly synthesized H4 [107]
	*NCAM1*	Neural cell adhesion molecule 1	Neural cell adhesion molecule, recently related with systemic lupus erythematosus [108]
	*CNTN1*	Contactin 1	Cell adhesion molecules implicated in myelinated axon organization [75]

## Data Availability

The original contributions presented in this study are included in the article/Supplementary Material. Further inquiries can be directed to the corresponding author.

## Supplementary Materials

The following supporting information can be downloaded at: https://www.mdpi.com/article/10.3390/nu17162678/s1. File S1: differential_analysis_promoter; File S2: differential_analysis_TSS; File S3: venn_AZA_As_BPHE30; File S4: venn_BPHE30_effect_on_As_and_AZA; Table S1: Number of genes resulting as differentially methylated (hyper- or hypo-methylated) in transcription start sites (TSSs) with the different cell (co-)treatments with respect to the untreated control cells (see Supplementary File S2 for the complete report).

## Abbreviations

5-AzaC	5-Azacytidine
AA	Antioxidant activity
As	Arsenic
BPHE	Bronte pistachio hydrophilic extract
CC	Cyanidin chloride
CCE	Cyanidin chloride equivalents
DD	Double-digested DNA
FW	Fresh weight
GA	Gallic acid
GAE	Gallic acid equivalents
MD	Mediterranean diet
MeSAP-PCR	Methylation-sensitive arbitrarily primed PCR
NGS	Next-generation sequencing
PAC	Total proanthocyanidin content
PKGs	Pathway-key genes
SD	Single-digested DNA
TE	Trolox equivalent
TPC	Total polyphenol content
TSSs	Transcription start sites
