# Supplementary material for: Methylomic Signature and Epigenetic Damage Modulation of Bronte Pistachio (Pistacia vera L.) Hydrophilic Extract in Differentiated CaCo-2 Cells
Source: Nutrients. 2025 Aug 19;17(16):2678. doi: 10.3390/nu17162678 (PMC12389270; doi:10.3390/nu17162678)
Supplement: Supplementary file 1 [file nutrients-17-02678-s001.zip › S2_differential_analysis_TSS.zip!/Supplementary Table S1.pdf]

| **Treatment vs. Untreated Control** | Total | Hyper-Methylated | Hypo-Methylated |
|---|---|---|---|
| BPHE30 | 276 | 225 | 51 |
| As | 149 | 112 | 37 |
| As+BPHE30 | 345 | 288 | 57 |
| AZA | 336 | 207 | 129 |
| AZA+BPHE30 | 336 | 207 | 129 |

**Supplementary Table S1.** Number of genes resulting as differentially methylated (hyper- or hypo-methylated) in transcription start sites (TSSs) with the different cell (co-)treatments with respect to the untreated control cells (see Supplementary Files S2 for the complete report).
